# A metabolic link between the astrocytic α2-Na/K ATPase and episodic paralysis

**DOI:** 10.1038/s42003-021-01729-7

**Published:** 2021-02-05

**Authors:** George Andrew S. Inglis

**Affiliations:** Communications Biology, https://www.nature.com/commsbio/

## Abstract

While loss-of-function mutations affecting the α2-Na/K ATPase are known to cause familial hemiplegic migraine, it is unclear how reduced protein activity could contribute toward migraine or paralysis observed in patients. A recent study from Sarah Smith and colleagues demonstrates that conditional deletion of the α2-Na/K ATPase in astrocytes can evoke episodic paralysis in mice, potentially due to altered metabolic processing of serine and glycine. By feeding juvenile α2-Na/K ATPase mutant mice a serine- and glycine-free diet, the authors are able to prevent the onset of episodic paralysis. This study suggests that loss of α2-Na/K ATPase in astrocytes may affect amino acid metabolism in the brain, ultimately leading to episodic paralysis.

Mutations affecting the α2-Na/K ATPase involved in astrocytic cation transport cause familial hemiplegic migraine, a severe neurological disorder marked by sporadic migraine and partial paralysis. Previous studies have noted that loss-of-function or hypomorphic α2-Na/K ATPase variants are associated with migraines and paralysis, though the mechanism underlying each of these phenotypes is still unclear.

A recent study^1^ led by Sarah Smith at the Washington University School of Medicine in St. Louis suggests that loss of α2-Na/K ATPase may trigger episodic paralysis through altered metabolic processing. The authors observed that conditional deletion of α2-Na/K ATPase in mouse astrocytes evoked spontaneous paralysis in homozygous mutants aged 30–40 days old, without any confounding neuronal death or seizures. Furthermore, these mice exhibit a correlation between episodic paralysis and spontaneous cortical spreading depression, a common electrophysiological trait observed in patients with migraine. From transcriptomic analysis of nascent and translated mRNAs in the cerebral cortex of homozygous mutants and controls, the authors note that mice lacking astrocytic α2-Na/K ATPase exhibit a significant increase in the expression of genes linked to metabolism of amino acids, particularly glycine and serine. Interestingly, metabolomic analysis of the cerebral cortex also revealed an increase in glycine and serine levels in the homozygous α2-Na/K ATPase mutants, relative to controls. By subsequently feeding the mice on a glycine- and serine-free diet at postnatal day 17 (prior to onset of paralysis), the authors were able to prevent episodic paralysis from occurring in homozygous mutants, as well as significantly improve motor coordination and survival.

This study sheds light on a potential role for metabolic dysfunction in episodic motor paralysis due to altered α2-Na/K ATPase expression, which may open the door to dietary interventions for patients. Furthermore, by integrating several sequencing datasets, the authors have generated a valuable resource for other groups interested in pathways altered by α2-Na/K ATPase dysfunction. Altogether, these results present an interesting mechanism that may influence future research on episodic neurological disorders.

## References

[1] Smith SE (2020). Astrocyte deletion of α2-Na/K ATPase triggers episodic motor paralysis in mice via a metabolic pathway. Nat. Commun..

